# NMR resonance assignments of the pathogenesis-related peach allergen Pru p 1.0101

**DOI:** 10.1007/s12104-018-9864-x

**Published:** 2018-12-12

**Authors:** Sebastian Führer, Simone Trimmel, Kathrin Breuker, Martin Tollinger

**Affiliations:** Institute of Organic Chemistry and Center for Molecular Biosciences Innsbruck (CMBI), University of Innsbruck, Innrain 80/82, 6020 Innsbruck, Austria

**Keywords:** NMR resonance assignment, TALOS + prediction, PR-10 protein, Cross-reactivity, Allergen

## Abstract

In Europe, Northern America, and China a large number of individuals are suffering from peach (*Prunus persica*) allergy caused by the protein Pru p 1. Immunologic reactions against this 17.5 kDa protein result from initial sensitization to the birch (*Betula verrucosa*) pollen allergen Bet v 1 and subsequent immunologic cross-reactivity of Bet v 1 specific antibodies. Allergic symptoms typically include severe itching, scratching of the throat, and rhino conjunctivitis. So far, experimental structural data for the peach allergen Pru p 1 are not available. In a first step towards the elucidation of the structure of this protein we assigned backbone and side chain ^1^H, ^13^C, and ^15^N chemical shifts of the naturally occurring isoform Pru p 1.0101 by solution NMR spectroscopy. Our chemical shift data indicate that this protein fold consists of seven β-strands separated by two short α-helices and a long C-terminal α-helix, which is in accordance with the reported crystal structure of Bet v 1. Our data provide the basis for determining the three-dimensional solution structure of this protein and to characterize its immunologic cross-reactivity on a structural basis.

## Biological context

In the northern hemisphere, individuals who are sensitized to birch pollen show IgE serum reactivity against the protein Bet v 1, the major birch pollen allergen, in about 90% of all cases (Ipsen and Lowenstein 1983; Moverare et al. 2002). Patients suffering from birch pollinosis can additionally develop allergies to fruits and nuts due to immunological cross-reactivity of Bet v 1 specific antibodies to proteins that are present in these kinds of food. Consumption of fruits and nuts then triggers oral allergic syndromes (OAS) in many cases, including swelling and itching of the tongue, throat and lips (Mari et al. 2005). Indeed, 30–50% of birch pollen allergic individuals show allergic reactions after consuming peaches (Geroldinger-Simic et al. 2011; Pastorello et al. 1994), a prevalence that is only exceeded by allergic reactions to apples and hazelnuts (Geroldinger-Simic et al. 2011).

Immunological cross-reactivity to birch pollen is related to the protein Pru p 1 that is present in peaches (Mills and Shewry 2004). Pathogens or stress can induce the expression of Pru p 1, which is believed to be involved in the general plant defense mechanisms (Fernandes et al. 2013). Pru p 1 is distinct from another allergen in peach, Pru p 3, a non-specific lipid transfer protein that provokes severe allergic reactions typically in the Mediterranean population (Fernandez-Rivas et al. 2003; Pastorello et al. 1999) and which is present in peaches in higher concentrations (Ahrazem et al. 2007). Like its homolog Bet v 1, Pru p 1 belongs to the group 10 of pathogenesis-related (PR) proteins, which have a size of about 17 kDa and consist of about 160 amino acid residues. The PR-10-fold comprises a seven-stranded antiparallel β-sheet (β1–β7) that is covered by a long C-terminal α-helix (α3) and two short α-helices (α1, α2) interrupting the β-sheet between strands β1 and the consecutive strands β2–β7. Pru p 1 is easily degraded and heat labile (Asero et al. 2001), which is in accordance with other PR-10 proteins. Three naturally occurring isoforms of Pru p 1 have been identified in the ‘Early Gold’ peach cultivar (Chen et al. 2008) and their efficacies of IgE-binding have been studied (Gao et al. 2016). The Pru p 1.0101 (DQ251187) and Pru p 1.0201 (KM350692) isoforms are present in the peach fruit and the Pru p 1.0301 (KM350693) allergen is present in the peach pollen and the leaves of the peach tree (Gao et al. 2016). Experimental structural data for Pru p 1 isoforms are not available to date and NMR resonance assignments have not been published. Here we present the solution NMR backbone and side-chain assignment of the recombinantly expressed isoform Pru p 1.0101. This particular isoform shares sequence identity of 59% with the sensitizing allergen Bet v 1.0101 (Gajhede et al. 1996). Sequence identity to the most prominent cross-reactive allergen in food, the major allergen from apple (*Malus domestica*) Mal d 1.0101, is considerably higher (82%) (Ahammer et al. 2017, 2016).

## Methods and experiments

### Sample preparation

The codon-optimized DNA of Pru p 1.0101 (GenBank nucleotide code DQ251187 and protein code ABB78006) was cloned into the expression vector pET28b (+) by the restriction enzymes NcoI and XhoI. Plasmid transformation was carried out in the *E. coli* strain BL21(DE3) Star (Invitrogen). A starter culture (100 mL) of Luria Bertani (LB) medium containing 25 µg/mL kanamycin was prepared by inoculation with one single bacterial colony and incubated at 37 °C and 220 rpm overnight. The cells of 50 mL of the starter culture were collected by centrifugation (2000×*g*) and resuspended in 1 L of M9 minimal medium supplemented with 25 µg/mL kanamycin and enriched with ^13^C_6_-d-glucose and/or ^15^NH_4_Cl (both Cambridge Isotope Laboratories). Incubation of the culture was carried out at 37 °C and 220 rpm until the cell density reached about 0.5 and induction of protein expression was induced by IPTG (isopropyl-β-d-1-thiogalactopyranosid, 1 mM). Incubation was performed for 3 h at 37 °C and afterwards the cells were harvested by centrifugation at 3440×*g* and 4 °C for 30 min. The cell pellets were resuspended in 25 mM imidazole, 0.1% Triton X-100, and 0.5 M urea. The cell suspension was shock-frozen in liquid nitrogen and stored at − 80 °C until usage. For Pru p 1 purification, the frozen cells were thawed and pre-treated with lysozyme (10 µg/mL) for 1 h on ice. Subsequently, DNAse I (1 µg/mL) was added and cells were lysed with a French Press. The lysate was centrifuged at 15,000×g and 4 °C for 40 min and loaded onto an anion exchange column (Resource Q 6 ml, GE Healthcare). Pru p 1.0101 was eluted with a sodium chloride gradient over 30 mL from 0 to 50% in 25 mM TrisHCl buffer (pH 7.5) at a flow rate of 2 mL/min. Fractions containing Pru p 1.0101 were collected and concentrated to 1–2 mL by centrifugation (Amicon Ultra 3 kDa MWCO, Merck Millipore). In the final step, the protein was loaded onto a size exclusion column (HiLoad 16/600 Superdex 75 prep grade, GE Healthcare) and eluted with 10 mM sodium phosphate buffer (pH 6.9) at a flow rate of 1 mL/min. Fractions containing Pru p 1.0101 were pooled and concentrated. For NMR spectroscopy, samples were supplemented with 10% D_2_O (v/v), yielding concentrations of 0.5 mM for ^15^N labeled and ^15^N/^13^C labeled Pru p 1.0101.

Expression and purification steps of Pru p 1.0101 were monitored by SDS–PAGE gel electrophoresis using 15% gels. The purified protein was analyzed by mass spectrometry using a 7 T Fourier-transform ion cyclotron resonance mass spectrometer (FT-ICR MS; Apex Ultra 70, Bruker Daltonics) with an electrospray ionization (ESI) source. The degree of isotope labeling for ^15^N and ^15^N/^13^C labeling was determined as 99.5 ± 0.1% and 98.8 ± 0.1%, respectively. In addition, the mass spectrometry data showed that in 97% of Pru p 1.0101 the N-terminal methionine was cleaved off.

### NMR spectroscopy

All NMR spectra were recorded at 25 °C on a 500 MHz Agilent DirectDrive 2 spectrometer equipped with a room temperature probe. A two-dimensional ^1^H-^15^N-HSQC and three-dimensional HNCACB, CBCA(CO)NH, HNCO, and HN(CA)CO experiments were performed for backbone resonance assignments. Side-chain assignments were obtained by using ^1^H-^13^C-HSQC and three-dimensional H(CCO)NH-TOCSY, (H)CC(CO)NH-TOCSY, ^1^H-^15^N-TOCSY-HSQC, ^1^H-^15^N-NOESY-HSQC, and ^1^H-^13^C-NOESY-HSQC experiments. Processing of the collected data was done with NMRPipe (Delaglio et al. 1995) and resonance assignment was performed using CcpNMR (Vranken et al. 2005) software.

### Assignments and data deposition

**Fig. 1:**
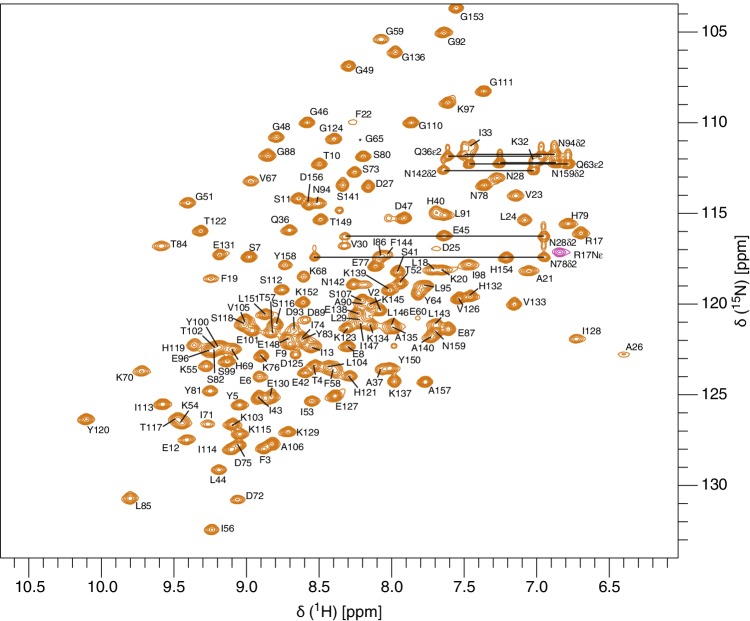
500 MHz ^1^H-^15^N-HSQC spectrum of Pru p 1.0101 (0.5 mM) in 20 mM sodium phosphate (pH 6.9), supplemented with 10% D_2_O, 25 °C. Assigned residues are indicated using single letter codes and horizontal lines indicate asparagine and glutamine NH_2_ side-chain resonances. The signal labeled by an asterisk indicates the position of the residue below the intensity cut-off. Resonance assignments are available online at the BMRB repository (Accession Number 27687)

In the ^1^H-^15^N-HSQC spectrum of Pru p 1.0101 (Fig. 1) we achieved an assignment for 142 of 151 non-proline residues corresponding to 94.0% completeness. Assignments of C^α^ and C^β^ resonances are 95.6% and 95.8% complete, respectively, while backbone C′ assignments are 94.3% complete. Side-chain C^γ^, C^δ^, and C^ε^ were assigned to 69.0%, 57.8%, and 45.0% completeness, respectively. In addition, 93.7% of H^α^ and 93.6% of H^β^ resonances along with 84.8%, 71.3%, and 61.9% of side-chain H^γ^, H^δ^, and H^ε^ resonances, respectively, were assigned. Full assignments of the side-chain amides (^1^H and ^15^N) of all five asparagine and two glutamine residues, along with the side-chain H^ε^/N^ε^ of the single arginine residue at position 17 were obtained. The chemical shift data of Pru p 1.0101 have been deposited at the Biological Magnetic Resonance Data Bank (http://www.bmrb.wisc.edu) with the BMRB accession number 27687.

**Fig. 2:**
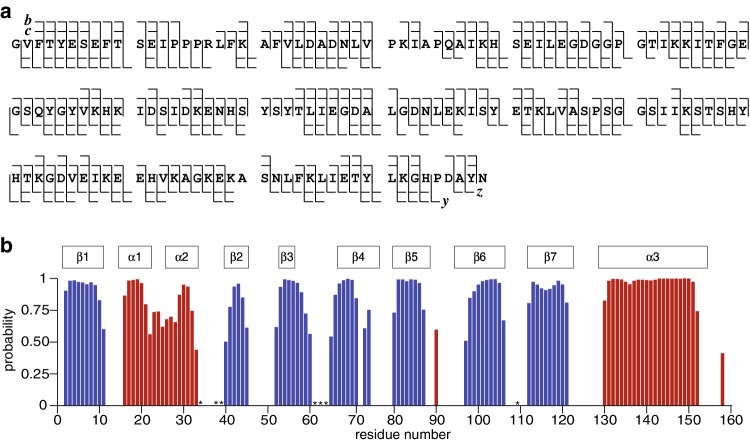
Primary and secondary structure of Pru p 1.0101 **a** MS/MS fragment map for the 159-residue protein Pru p 1.0101 (measured mass for most abundant isotopic signal: 17515.97 Da, calculated: 17515.99 Da) illustrating 96% sequence coverage; *c, z*^*•*^ and *b, y* fragments were produced by electron capture dissociation (ECD) and collisionally activated dissociation (CAD), respectively. **b** TALOS+ prediction of Pru p 1.0101 secondary structure elements based on backbone H^N^, N, C′, C^α^, and C^β^ chemical shifts. The secondary structure probabilities (red, α-helices; blue, β-strands) are plotted against the residue numbers. Residues for which backbone amide NH resonance assignments are not available are indicated by asterisks. Secondary structure elements of Bet v 1.0101 (PDB: 4A88) are indicated

The amino acid sequence of Pru p 1.0101 was verified by MS/MS (Fig. 2a), showing 96% sequence coverage. Secondary structure elements of Pru p 1.0101 were obtained by the TALOS+ prediction software (Shen et al. 2009) using the H^N^, N, C′, C^α^, and C^β^ backbone chemical shifts as input (Fig. 2b). Our data shows that this protein comprises seven β-strands (β1–β7), two short α-helices (α1 and α2) and a long C-terminal α-helix (α3), which is consistent with the PR-10 fold found in crystallographic and NMR spectroscopic investigations of Bet v 1.0101 and Mal d 1.0101, respectively (PDB: 4A88 and 5MMU). In addition, the segment between β5 and β6 and the C-terminal region after α3 appear to have a moderate propensity for α-helical structure. The NMR resonance assignment of Pru p 1.0101 obtained here provides the basis for determining the three-dimensional solution structure of this important protein in the future.
